# Sublethal effects of insecticides on the reproduction traits of insecticide-resistant *Anopheles gambiae* sensu lato mosquitoes from Tiassalé, Côte d’Ivoire

**DOI:** 10.1186/s13071-026-07431-z

**Published:** 2026-06-05

**Authors:** Koffi Romiald Kouame, Allassane Foungoye Ouattara, Katherine Gleave, Behi Kouadio Fodjo, Julien Bi ZahouliZahouli, Ruth Marie Adjoua Kouame, Graham John Small, Janneke Snetselaar, Benjamin Guibehi Koudou

**Affiliations:** 1 Centre Suisse de Recherches Scientifiques en Côte d’Ivoire, Abidjan, Côte d’Ivoire; 2 UFR Sciences de La Nature, Université Nangui Abrogoua, Abidjan, Côte d’Ivoire; 3 Innovative Vector Control Consortium, Liverpool, UK; 4 Centre d’Entomologie Médicale Et Vétérinaire, Université Alassane Ouattara, Bouaké, Côte d’Ivoire; 5 Université Alassane Ouattara, Bouaké, Côte d’Ivoire

**Keywords:** Malaria, Mosquito, Sublethal, Broflanilide, Chlorfenapyr, Deltamethrin, Life-history trait, Insecticide resistance, Vector control

## Abstract

**Background:**

Although exposure of *Anopheles* mosquitoes to insecticide-treated vector control tools is not always lethal, the sublethal effects of these insecticides on their life-history traits remain poorly explored. Therefore, this study examined the impact of sublethal exposure to broflanilide, chlorfenapyr and deltamethrin on longevity; blood meal acceptance; viable egg production; and the proportion of females with viable eggs in insecticide-resistant *An. gambiae* s.l. populations from Tiassalé, Côte d’Ivoire.

**Methods:**

The sublethal effects of broflanilide, chlorfenapyr and deltamethrin were assessed on 3–5-day-old adult female *An. gambie s.l.* from the field collected Tiassalé strain (F_0_), using the insecticide-susceptible Kisumu strain as reference, following standardized World Health Organization (WHO) bottle bioassay procedures. For each insecticide, the lethal dose causing 20% mortality (LD_20_) was determined through dose–response analysis and then subsequently applied in treated bottles. Post-exposure, longevity, blood-feeding success, egg viability and the proportion of ovipositing females were recorded. Comparisons were made between the control and LD_20_ treatment groups.

**Results:**

Sublethal exposures produced distinct effects depending on both the mosquito strain and the insecticide type. Broflanilide significantly reduced longevity in the Tiassalé population (*P* < 0.0001), whereas chlorfenapyr and deltamethrin had stronger effects on the Kisumu strain. Deltamethrin exposure notably impaired blood-feeding behaviour, reducing feeding success by 25.5% in Kisumu and 19.5% in Tiassalé. Reproductive parameters were also affected: both chlorfenapyr and broflanilide reduced viable egg production and the proportion of females producing viable eggs in both strains. In contrast, deltamethrin showed a more limited impact on reproduction. In the Tiassalé strain, the proportion of females producing viable eggs dropped to 41.5% following chlorfenapyr exposure, compared with 65.7% in unexposed individuals.

**Conclusions:**

These findings demonstrate that sublethal doses of insecticides can adversely affect reproductive success and blood-feeding behaviour in both susceptible and resistant *Anopheles* mosquitoes, potentially reducing their vectorial capacity. This study underscores the need to systematically account for sublethal effects when evaluating insecticides in vector control programs, particularly in settings where resistance is widespread among malaria vectors.

**Graphical Abstract:**

**Figure:**
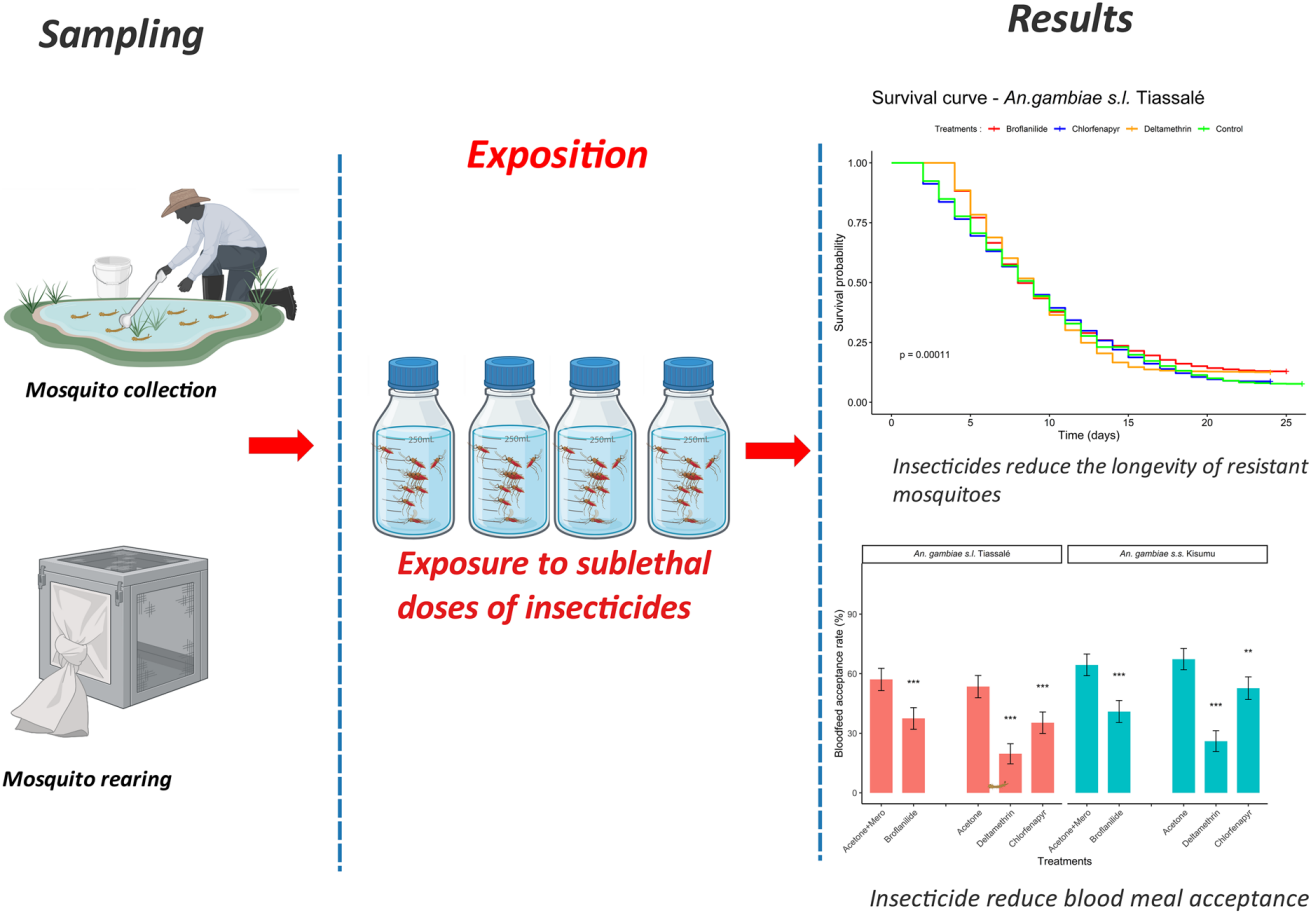

## Background

Malaria is caused by *Plasmodium* parasites transmitted to humans through the bites of infected *Anopheles* mosquitoes. For transmission to occur, the parasite must undergo a complex developmental cycle within the mosquito, progressing through several stages before becoming infectious to humans. Therefore, limiting the abundance of mosquitoes or hindering their ability to support parasite development by affecting their life-history traits can help reduce malaria transmission. In malaria endemic areas, insecticide-treated nets (ITNs) and indoor residual spraying (IRS) remain the cornerstone of vector control and have achieved considerable success. However, the effectiveness of these strategies has been compromised by the emergence of insecticide resistance in mosquito populations, particularly against pyrethroids, the main components of chemical control tools [1–3]. This resistance arises from multiple factors, including strong selective pressure on mosquito populations from widespread use of insecticides in both agriculture and public health. Furthermore, reduced contact time between mosquitoes vector control interventions can limit insecticide exposure, facilitating the survival and spread of resistant populations [4, 5]. Over recent years, numerous studies have investigated the physiological responses of different mosquito species following sublethal exposure to insecticides used in public health or under evaluation for use in vector control [6–9]. Sublethal effects on mosquito–parasite interactions have also been demonstrated, influencing vectorial capacity in both laboratory and field populations of resistant *Anopheles* strains in Burkina Faso and Uganda [8, 10, 11]. Research in Côte d`Ivoire on multi-resistant wild *Anopheles gambiae* sensu stricto populations have shown high variability in the effects of sublethal doses on mosquito vectorial capacity [12, 13].

The World Health Organization’s (WHO) Global Plan for Insecticide Resistance Management in Malaria Vectors (GPIRM) recommends rotating insecticides with differing modes of action and developing novel active ingredients for which there is no known resistance as part of its strategy [14].This strategy also includes repurposing compounds that were originally designed for use in agriculture, such as broflanilide (Tenebenal™) and chlorfenapyr [14–16]. Broflanilide is a meta-diamide insecticide that acts by targeting the glycine residue located at position 3 (G3′) in the third transmembrane domain (TMD3) of the RDL subunit of the insect GABA receptor. This allosteric interaction blocks inhibitory nerve signal transmission, leading to neuronal hyperexcitation, convulsions and death of the insect. Its binding site differs from those of conventional GABA-modulating insecticides, providing efficacy against *Anopheles* species resistant to pyrethroids, with no evidence of cross-resistance [15, 17]. Broflanilide induces delayed mortality, atypically manifesting within 72 h post-exposure and demonstrates prolonged residual efficacy in IRS trials [15, 17, 18]. Chlorfenapyr is a pyrrole insecticide with a non-neurological mode of action. It disrupts mitochondrial oxidative phosphorylation, depleting cellular energy and resulting in death of the mosquito. Studies have shown its effectiveness against several species resistant to pre-existing classes of insecticide, with IRS treatments demonstrating residual activity lasting up to 34 weeks in both laboratory and field trials [19, 20]. The absence of cross-resistance to existing insecticides strengthens the value of chlorfenapyr for resistance management, despite its slower mode of action, with mortality typically increased 48–72 h after exposure [21–23].

While several studies have investigated the sublethal effects of standard pyrethroid insecticides [24–27], few have explored how sublethal exposure to new-generation insecticides affects mosquito life-history traits and potential pathogen transmission. WHO guidelines currently prioritize mortality and blood-feeding inhibition as key indicators of insecticide efficacy; however, sublethal effects can also significantly influence disease transmission dynamics [28]. Previous research has revealed that even brief exposure to deltamethrin or chlorfenapyr reduces blood feeding and longevity, thereby limiting vectorial capacity independent of direct lethality [29, 30]. Therefore, evaluation of insecticidal efficacy should incorporate both lethal and sublethal responses [31, 32]. Residual effects on traits such as longevity, blood feeding success, viable egg production and proportion of females with viable eggs needs to be further explored with evidence-based data. This approach can help improve understanding around how the use of vector control tools can generate sustained effects that continue to shape transmission dynamics after direct insecticidal potency declines. In this study, we evaluated the sublethal effects of broflanilide, chlorfenapyr and deltamethrin on key reproductive parameters in the insecticide-resistant Tiassalé and the insecticide-susceptible Kisumu strains of *Anopheles gambiae*. These parameters included longevity, blood feeding success, viable egg production and proportion of females with viable eggs [33]. We hypothesized that sublethal exposure would lead to reduced values across these parameters compared with untreated controls.

## Methods

### Study areas

**Fig. 1:**
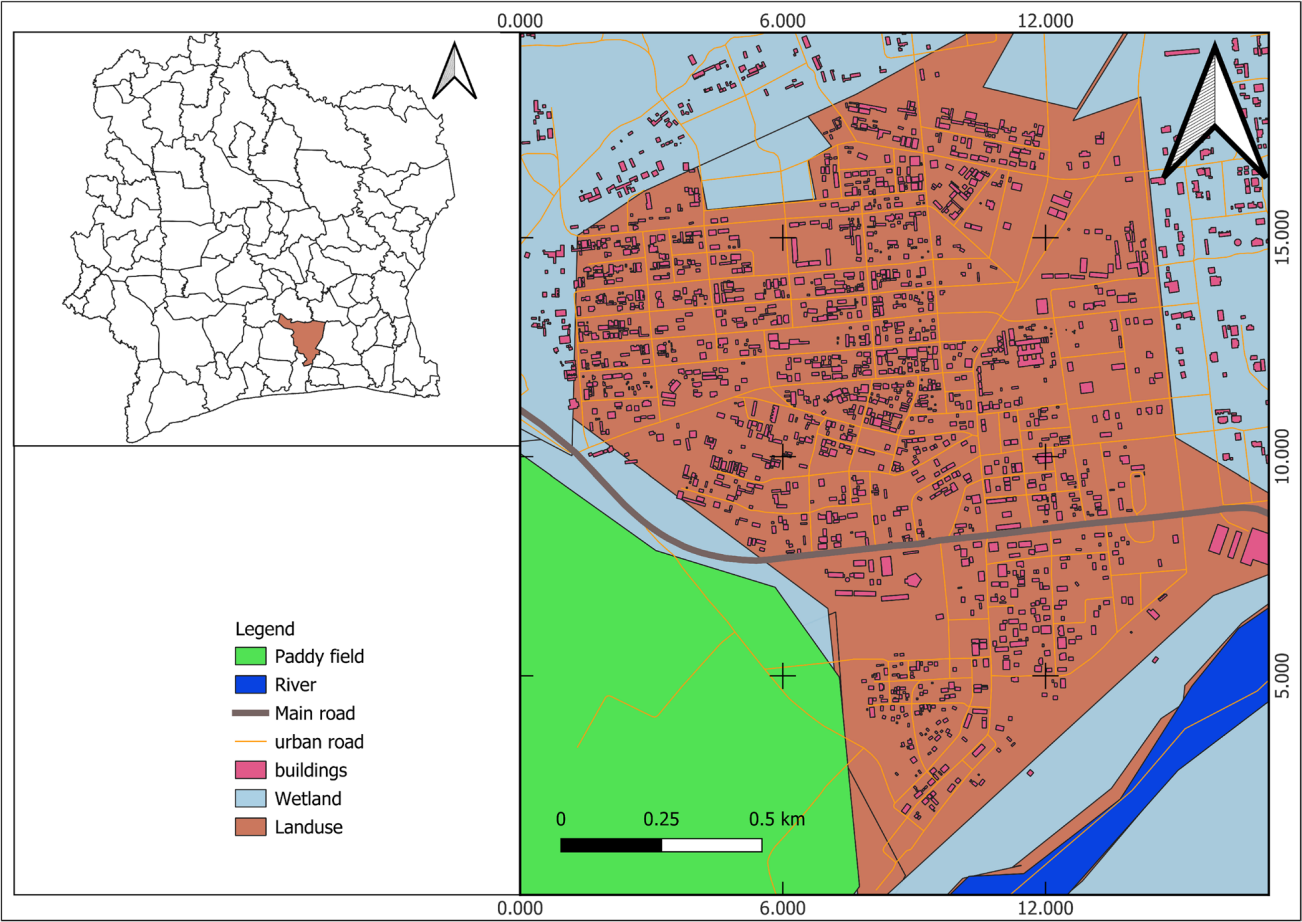
Map showing the study area in Tiassalé, southern Côte d’Ivoire

The study was conducted in Tiassalé (5° 54′ N, 4° 50′ W), southern Côte d’Ivoire (West Africa) from October 2023 to September 2024. Tiassalé is close to the Bandama River, which plays a crucial role in the development of local agriculture, facilitating the irrigation of alluvial lowlands, which are heavily used for rice cultivation (Fig. 1). The irrigated rice fields and crops are located near several urban and rural neighbourhoods (Tiassalé and Tiassalékro) and have created ecological conditions conducive to the development of mosquitoes, mainly *Anopheles* malaria vectors [34, 35]. These rice fields and crops are regularly treated with pesticides that have contributed to insecticide resistance in the local *Anopheles* vectors. Tiassalé has therefore became an area where the country’s main malaria vector, *An. gambiae*, is exceptionally resistant to all insecticide classes [36, 37]. This has made the Tiassalé locality and *An. gambiae* populations, respectively, a reference site and strain for both research on vector resistance and trials for testing the efficacy of new vector control tools (e.g. ITNs and IRS) in the fight against malaria.

This map was generated using Quantum Geographic Information System (QGIS) software version 3.38.8-Grenoble (https://www.qgis.org/), with the basemap shapefile sourced from the Database of Global Administrative Areas (GADM, https://gadm.org/; license: https://gadm.org/license.html). The geographical layers representing buildings, the road network and wetlands come from OpenStreetMap (https://www.openstreetmap.org/). All spatial data were imported in shapefile format and projected in the WGS84 coordinate system (EPSG:4326).

### Mosquitoes

The laboratory-reared, susceptible *An. gambiae* s.s. Kisumu strain originates from the Kisumu region of Kenya and is known to be fully susceptible to the predominant insecticide classes [38–40]. The wild *An. gambiae* sensu lato mosquitoes were collected as larvae in the rice fields near the experimental station of Tiassalé. Molecular species identification was not performed on the mosquitoes used in this study; however, previous molecular surveys conducted at the Tiassalé experimental station consistently showed a strong dominance of *Anopheles coluzzii* (> 95%), with a study conducted in 2023 showing 100% *An. coluzzii* (*n* = 50) [41, 42]. Therefore, the study results are interpreted at the *An. gambiae* s.l. level. The local populations of *An. gambiae* s.l. are multi-resistant to many insecticides [41]. Very high mutational frequencies (i.e. *kdr*, 0.83), target-site resistance genes (i.e. *Ace-1R*: 0.44) and metabolic resistance genes (i.e. cytochrome P450s) have been detected in the *An. gambiae* s.l. Tiassalé populations [41]. In addition, overexpression of several CYP6 P450 genes and the *COEAE6G* carboxylesterase gene has been detected [37, 41–43].

The Kisumu and Tiassalé strains were reared to adulthood at the CSRS insectary (GLP accreditation) located in Tiassalé. Larvae were reared in trays containing 120–150 larvae in 700–800 mL of tap water and fed daily with Tetramin Baby Fish (TetraMin® Tropical Flakes, Tetra®, Blacksburg, VA, USA). The larvae are reared under standardized and controlled conditions (i.e. 25 ± 2 °C, 75 ± 10% relative humidity (RH), and light–dark 12 h: 12-h photoperiod). Adults were provided access to a 10% sugar solution ad libitum prior to use in experiments. For the Tiassalé strain, all mosquitoes used were F_0_ generation adults aged 2–5 days derived from larvae directly collected from rice field.

### Insecticides

The insecticides used were broflanilide (technical grade, 99.1% purity, supplied by Mitsui Chemical Crop & Life Solutions, Inc., Tokyo, Japan), chlorfenapyr (technical grade, 99.3% purity, from Sigma-Aldrich) and deltamethrin (technical grade, 98.6% purity, from Bayer AG). The stock solutions were prepared by dissolving the broflanilide in acetone plus the surfactant Mero (concentration 800 ppm; 81% rapeseed oil methyl ester; Bayer AG, Crop Science Division), and chlorfenapyr and deltamethrin in acetone alone.

### Determination of the sublethal dose of insecticides (LD_20_)

The lethal dose expected to cause 20% mortality (LD_20_) was determined for each of the three insecticides tested. The LD_20_ dose was selected, as it provided an appropriate balance between inducing measurable mortality to represent significant selective pressure while retaining sufficient survivors for the assessment of life-history parameters. Concentration ranges were prepared for each insecticide on the basis of data from previous studies to determine the LD_20_. The concentrations were 0.01, 0.05, 0.1, 0.25, 0.5, 1, 2.2, 4.6 and 10 µg/bottle for broflanilide; 3, 5, 8, 10, 20, 30, 40, 50, 60, 70, 80, 90 and 100 µg/bottle for chlorfenapyr; and 0.0156, 0.0313, 0.0625, 0.125, 0.25, 0.5, 1, 3, 5, 7, 8, 9, 10, 11 and 12.5 µg/bottle for deltamethrin [38, 44, 45].

LD_20_ was determined using bottle bioassays, performed according to the WHO standard operating procedure for testing insecticide susceptibility of adult mosquitoes [45]. Six 250 mL bottles (four test and two control) with labelled caps were coated with either 1 mL of insecticide solution for the test bottles and 1 mL of acetone (for the negative control bottles used in chlorfenapyr and deltamethrin bottle bioassays) or acetone + Mero at a final concentration of 800 ppm (89 µL of Mero were added to 100 mL of acetone for the negative control bottles used in broflanilide bottle bioassays). After evenly coating the bottles and lids, the bottles were opened and wrapped in aluminium foil to prevent photodegradation of insecticides and were dried in the dark for 24 h. Before the bioassays, unfed adult female mosquitoes, aged 3–5 days, were kept in the test room for 1 h to acclimatize. They were then introduced into all the control and test bottles at a rate of 25 mosquitoes per bottle.

After 1 h of exposure in bottles, mosquitoes were transferred using a mouth aspirator into labelled disposable cups covered with a net and the number of mosquitoes knocked down was recorded. A piece of cotton soaked in a 10% sugar solution was placed on top of the net of each cup. All mosquito testing and holding was carried out under standardized and controlled conditions described above. Post-exposure, mortality was recorded after 24 h for deltamethrin, and at 24, 48 and 72 h for broflanilide and chlorfenapyr because of their delayed action [17, 45]. Mortality after 24 h was used to calculate the LD_20_ for deltamethrin and 72 h for chlorfenapyr and broflanilide. After the mortality assessment, the surviving mosquitoes were removed and killed in a freezer and disposed of. Bottle bioassays were carried out three times and on different dates for each of the above concentrations of the three insecticides and for each of the mosquito strains. The impact of exposure in bottles to the estimated LD_20_ was then assessed on the longevity, blood meal acceptance, viable egg production and proportion of females with viable eggs of the *An. gambiae* Kisumu and Tiassalé strains.

### Sublethal exposure bioassays in WHO bottles

Each sublethal exposure was carried out according to the method and conditions used above, on both mosquito strains. Sublethal observations and evaluations were then performed on the basis of mosquito longevity, blood meal acceptance, viable egg production and proportion of females with viable eggs.

### Effect of exposure to the LD_20_ on adult longevity

The effects of sublethal exposure on mosquito longevity were assessed by transferring mosquitoes that survived insecticide exposure to mosquito cages and providing a 10% sugar solution, with no opportunity for a blood meal. The mortality of each group of mosquitoes was recorded daily until 100% mortality was observed.

Each experimental series consisted of four bottles containing 25 adult female mosquitoes each, for a total of 100 mosquitoes per series. Three independent replicates were performed for each insecticide and each strain. The same procedure was followed for untreated controls.

### Effect of exposure to the LD_20_ on blood meal acceptance

Blood meal acceptance was calculated as the proportion of females accepting a blood meal after exposure to an insecticide, in comparison with mosquitoes exposed to control [46]. We performed exposures using the same bottle bioassay method as described above. Four batches of 25 females aged 2–3 days, i.e., 100 mosquitoes per series, were deprived of sugar for 6 h before the blood meal acceptance assay. Mosquitoes were then exposed for 1 h and then moved to mosquito cages. The same process was followed for test and control mosquitoes. All mosquitoes were offered a 30-min blood meal on Guinea pigs (*Cavia porcellus*). The abdomens of females were observed to separate those which had partially fed and those which had fed to repletion [46]. This experiment was repeated three times, representing a total of 300 females per insecticide and per strain.

### Effect of exposure to the LD_20_ on viable egg production and proportion of females with viable eggs

This experimental design evaluated reproductive outcomes following sublethal exposure in mated females and did not allow for the separation of effects on mating success, oogenesis or egg development.

In this study, the proportion of females with viable eggs was defined as the proportion of females producing at least one mature (Christopher stages IV–V) egg, while viable egg production referred to the mean number of viable eggs per female in accordance with the WHO tunnel test guidelines (2023) [33, 47, 48]. The effect of sublethal exposure to broflanilide, chlorfenapyr and deltamethrin was assessed using the protocol described in Rigby et al. [46] and adapted to that described by Myers et al. [49] for ovary dissection methods.

Newly emerged males (50) and females (100) of each mosquito strain were placed together in 30 × 30 × 30 cm cages for 5–8 days to achieve a high percentage of mated females. Bottle bioassays were performed as above and after 24 h mosquitoes were offered an opportunity to feed on a Guinea pig for 30 min. Blood-fed females from treated and control groups were isolated in cups covered with untreated net, provided with 10% sugar solution and held for 72 h.

After 72 h, mosquito ovaries were dissected under a stereo microscope at 10× magnification [49]. Eggs were observed under a light microscope at 4× and 10× magnification to determine their stage of development [49].

### Statistical analysis

Data were transcribed from raw data sheets into Microsoft Excel and analysed in R (version 4.5.1; R Foundation for Statistical Computing, Vienna, Austria). Data quality was ensured through double manual verification and automated consistency checks, allowing the detection and correction of entry errors. Descriptive statistics were used to summarize cumulative mortality, blood-feeding rates, viable egg production and the proportion of females producing viable eggs.

Dose–response data from WHO bottle bioassays were analysed using log-probit regression to estimate LD_20_ values for each insecticide, with corresponding 95% confidence intervals (CI). Mosquito longevity was examined using survival analysis: Kaplan–Meier curves were generated for each treatment and compared with log-rank tests [46], while Cox proportional hazards models provided hazard ratios (HR), accounting for mosquito strain and estimating the relative risk of mortality over time.

Blood-feeding behaviour was analysed separately for each strain using binary logistic regression models, with exposure status as the main predictor and the control group as reference. Results were expressed as odds ratios (OR) with 95% CI and associated *P*-values. Differences between treatments were further explored using estimated marginal means with Tukey-adjusted post hoc comparisons.

Reproductive outcomes were analysed using complementary approaches. Mean viable egg production was described with 95% CI based on normal approximation. The proportion of females producing viable eggs was estimated using exact binomial (Clopper–Pearson) intervals. As egg production data did not follow a normal distribution [Shapiro–Wilk test, *W* = 0.83, (*df*) = 3, *P* < 0.0001], group comparisons were performed using the non-parametric Kruskal–Wallis test. For the proportion of egg-laying females, associations with insecticide exposure were first assessed using a Chi square test of independence. When significant, pairwise comparisons between treatments were conducted using proportion tests (prop.test command in R), with Holm adjustment applied to control for multiple testing.

## Results

### Sublethal dose (LD_20_)

LD_20_ varied considerably according to the mosquito strains and insecticides used. LD_20_ of broflanilide was 0.09 (95% CI = 0.04–0.15) µg/bottle for the Tiassalé strain and 0.03 (95% CI = 0.01–0.05) µg/bottle for the Kisumu strain (three times higher in Tiassalé). For chlorfenapyr, LD_20_ was 15.10 (95% CI = 12.70–17.30) µg/bottle in the Tiassalé strain and 8.33 (95% CI = 6.18–10.40) µg/bottle in the Kisumu strain (1.8 times higher in Tiassalé). LD_20_ of deltamethrin was 10.60 (95% CI = 7.62–13.60) µg/bottle for the Tiassalé strain and 0.02 (95% CI = 0.02–0.03) µg/bottle for the Kisumu strain (530 times higher in Tiassalé) (Tables 1, 2).

**Table 1:** Insecticide sublethal doses (LD_20_) with their CIs estimated for the Kisumu and Tiassalé strains of *Anopheles gambiae* mosquitoes

Mosquito	Broflanilide LD_20_ (95% CI)	Chlorfenapyr LD_20_ (95% CI)	Deltamethrin LD_20_ (95% CI)
Tiassalé strain	0.09 (0.04–0.15)	15.10 (12.70–17.30)	10.60 (7.62–13.60)
Kisumu strain	0.03 (0.01–0.05)	8.33 (6.18–10.40)	0.02 (0.02–0.03)

LD_20_ is a dose of insecticide estimated to kill 20% of exposed mosquitoes. The unit of LD_20_ is microgram per bottle

**Table 2:** The results of all the concentrations used to determine the LD_20_

Strain	Species: *An. gambiae*										
Kisumu											
Deltamethrin											
Dose (µg/bt)	0.0156	0.0313	0.0625	0.125	0.25	0.5	1	5	7	9	12.5
Total tested	299	300	300	302	297	297	296	299	300	300	300
Death after 24 h	42	80	140	178	253	287	291	299	300	300	300
24-h mortality (%)	14.0	26.7	46.7	58.9	85.2	96.6	98.3	100	100	100	100
Chlorfenapyr											
Dose (µg/bt)	3	8	10	20	30	50	80	90	100		
Total tested	281	285	294	291	283	298	293	295	298		
Death after 72 h	12	60	85	139	200	270	293	295	298		
72-h mortality (%)	4.3	21.1	28.9	47.7	70.7	90.6	100	100	100		
Broflanilide											
Dose (µg/bt)	0.01	0.05	0.1	0.25	0.5	1	2.2	4.6	10		
Total tested	295	300	292	300	300	300	299	300	300		
Death after 72 h	47	65	125	290	300	300	299	300	300		
72-h mortality (%)	15.9	21.7	42.8	96.7	100	100	100	100	100		
Tiassalé											
Deltamethrin											
Dose (µg/bt)	1	3	5	7	8	9	10	11	12.5		
Total tested	297	300	300	297	300	300	300	300	300		
Death after 24 h	5	6	13	18	25	36	50	64	116		
24-h mortality (%)	1.7	2	4.3	6.1	8.3	12	16.7	21.3	38.7		
Chlorfenapyr											
Dose (µg/bt)	5	10	20	30	40	50	60	70	80	100	
Total tested	293	297	303	312	304	297	288	297	290	302	
Death after 72 h	13	29	100	112	155	160	183	215	211	250	
72-h mortality (%)	4.4	9.7	33	35.9	51	53.9	63.5	72.4	72.8	82.8	
Broflanilide											
Dose (µg/bt)	0.01	0.05	0.1	0.25	0.5	1	2.2	4.6	10		
Total tested	295	300	297	299	296	302	295	298	300		
Death after 72 h	30	44	47	120	153	234	262	298	300		
72-h mortality (%)	10.2	14.7	15.8	40.1	51.7	77.5	88.8	100	100		

*bt* bottle

### Effect on longevity

**Fig. 2:**
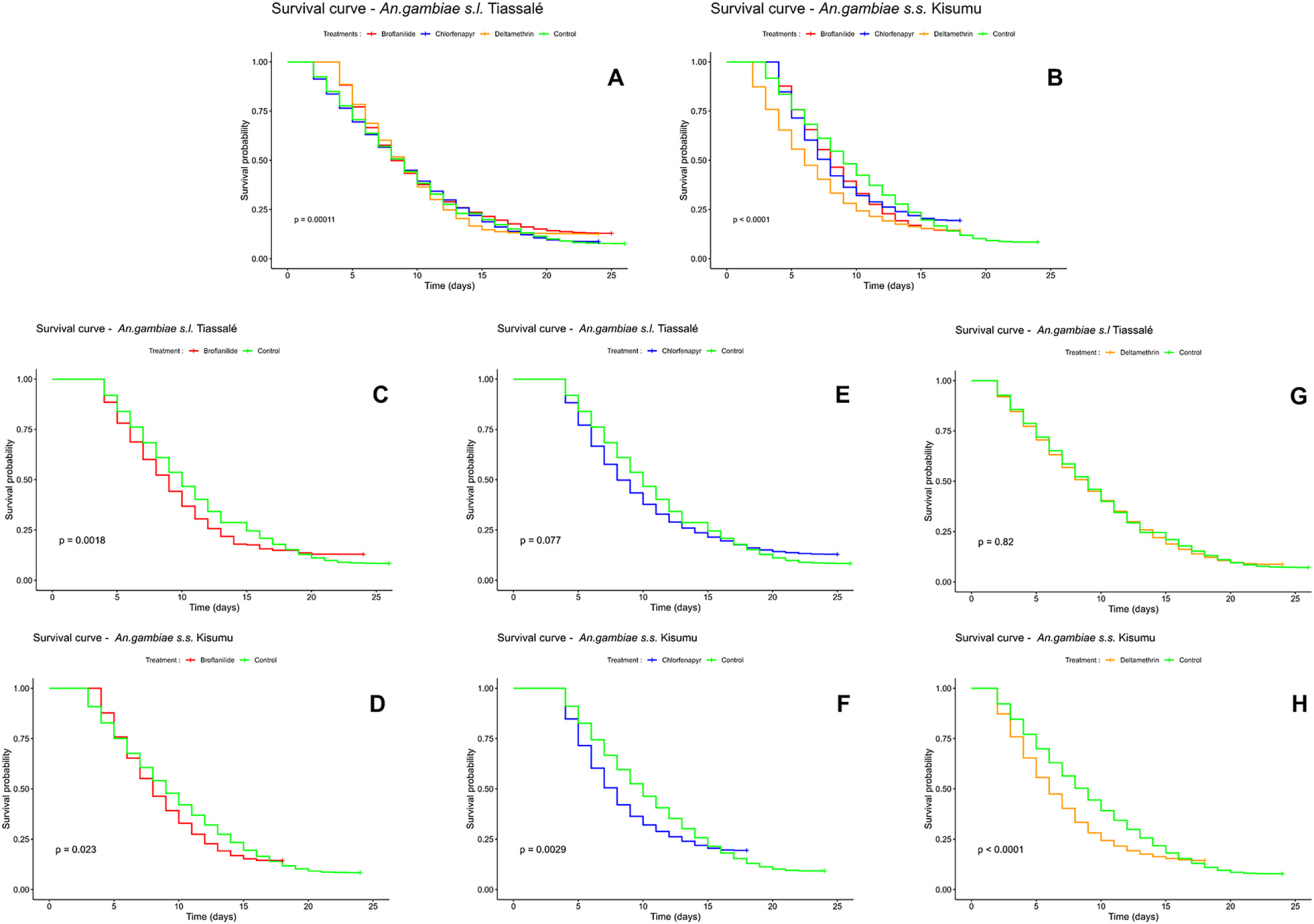
Daily survival curves of insecticide-resistant Tiassalé strains and the insecticide-susceptible Kisumu strains of *Anopheles gambiae* mosquitoes following exposure to the LD_20_ of broflanilide, chlorfenapyr and deltamethrin insecticides. Tiassalé strain survival curves: **A** all insecticides, **C** broflanilide, **E** chlorfenapyr, and **G** deltamethrin; Kisumu strain survival curves: **B** all insecticides, **D** broflanilide, **F** chlorfenapyr, and **H** deltamethrin

A simultaneous observation of daily mortality for the four treatments (three insecticides + one control) showed that there was a significant overall difference between at least two survival probabilities for each strain (*P* < 0.001). The Kaplan–Meier analysis showed *P* < 0.001 on the survival curves, with the survival curve (probability of survival) for mosquitoes exposed to uncoated bottles being significantly higher than for the other insecticide-treated bottles for both Tiassalé strains (*P* = 0.00052) and Kisumu strain (*P* < 0.0001) over observation time (Fig. 2A, B).

#### Broflanilide

The log-rank test showed a significant effect of the sublethal dose of broflanilide analysis on the longevity of Tiassalé strain (*P* < 0.0001) (Fig. 2C). Mosquitoes of the Tiassalé strain in the control bottles were 47.5% less likely to die on a given day than those exposed in the bottles treated with broflanilide [Cox model, *P* < 0.001, hazard ratio (HR) = 0.5, CI = 0.5–0.6]. Similar results were found for the Kisumu strain (Cox model, *P* = 0.031, HR = 0.9, CI = 0.9–1.0) (Fig. 2D).

#### Chlorfenapyr

Chlorfenapyr did not show a significant impact on the longevity of Tiassalé strain mosquitoes compared to mosquitoes exposed to acetone (control) bottles (*P* = 0.077) (Fig. 2E). Reduction in mosquito mortality risk between individuals exposed and unexposed to chlorfenapyr on the Tiassalé strain is very low and not significant (Cox model, *P* = 0.09, HR = 0.9, 95% CI = 0.9–1.0). By contrast, the susceptible Kisumu strain showed a statistically significant difference in survival distributions between the exposed and unexposed arms (log-rank test, *P* = 0.0029) (Fig. 2F). Additionally, there was a statistically significant reduction (10.5%) in mortality risk in the control groups (Cox model, *P* = 0.003, HR = 0.9, 95% CI = 0.8–1.0).

#### Deltamethrin

Mortality recorded beyond 24-h post-exposure to deltamethrin LD_20_ revealed differences in mosquito longevity based on the strain and the exposure type (insecticide-treated versus control). Kaplan–Meier survival curves indicated that the longevity of *An. gambiae* s.l. Tiassalé mosquitoes was not significantly affected (log-rank test, *P* = 0.82) by exposure to the LD_20_ of deltamethrin compared with unexposed mosquitoes (Fig. 2G). Moreover, the reduction in mortality risk was only 1.1% between these two exposure types. The mortality risk of Tiassalé mosquitoes exposed to control bottles was similar to those exposed to LD_20_ for deltamethrin (Cox model, *P* = 0.653, HR = 1.0, 95% CI = 0.9–1.0).

However, a significant difference in sublethal deltamethrin exposure in mosquito longevity was observed with the Kisumu strain (log-rank test, *P* < 0.0001) (Fig. 2H). Kisumu mosquitoes exposed to control bottles had 18.0% higher probability of survival than those exposed to the LD_20_ for deltamethrin (Cox model, *P* < 0.001, HR = 0.8, 95% CI = 0.8–0.9).

### Effect on blood-meal acceptance

**Fig. 3:**
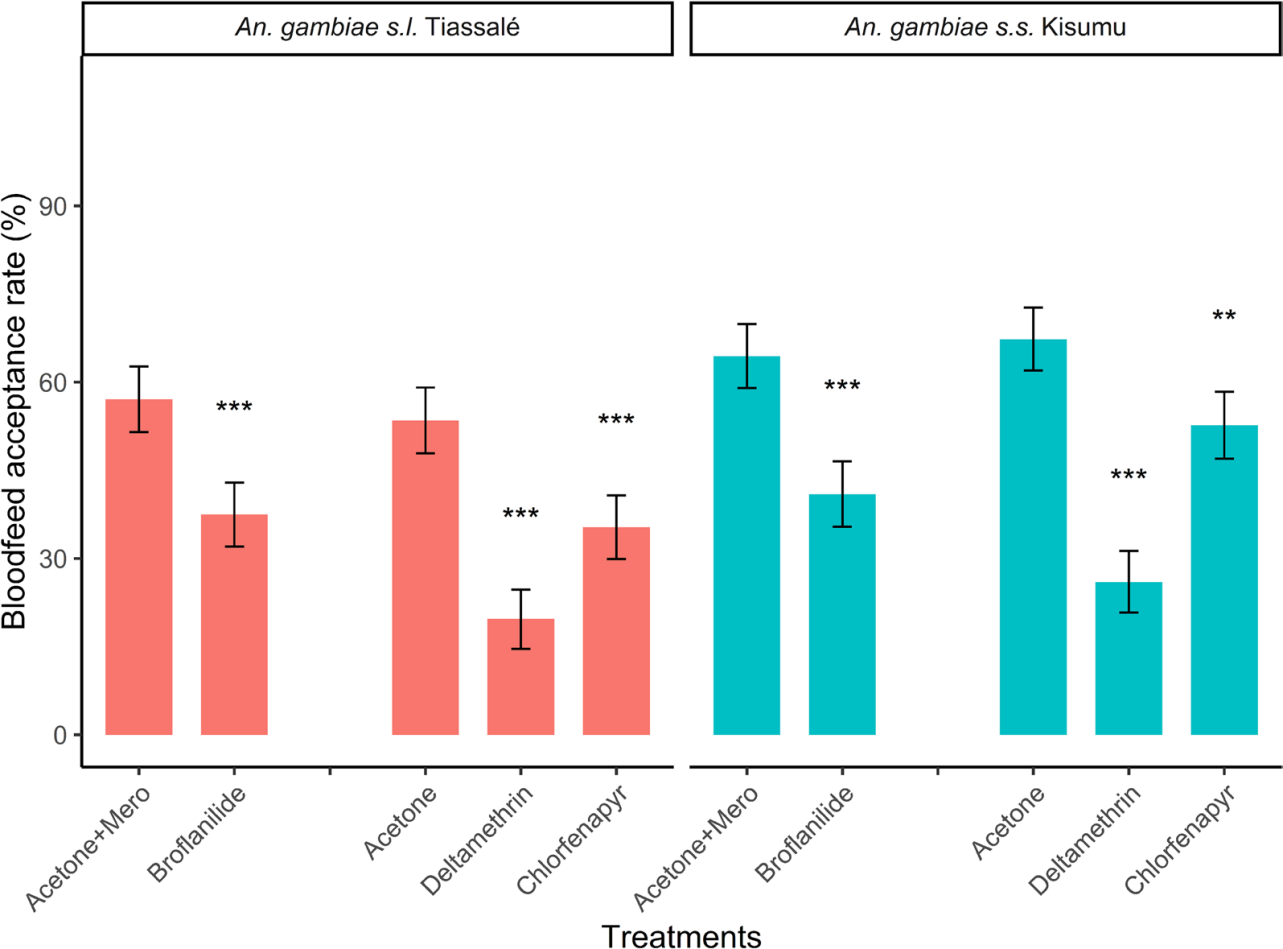
Blood-meal acceptance rate of Tiassalé and Kisumu strains of *Anopheles gambiae* mosquitoes exposed to sublethal doses of deltamethrin, chlorfenapyr or broflanilide [^**^significant at 1% level (*P* < 0.01); ^***^significant at 0.1% level (*P* < 0.001) compared with its control]

The proportion of blood-fed mosquitoes was higher in control bottles than in treatment bottles for both Tiassalé strain (53.0%, 95% CI = 49.3–60.4) and Kisumu strain (67.5%, 95% CI = 62.0–72.7) (Fig. 3). The lowest blood meal acceptance rates were observed in mosquitoes pre-exposed to the sublethal dose of deltamethrin for the Tiassalé strain (19.5%, 95% CI = 4.6–24.7%). The acceptance rate of the Kisumu strain after exposure to the sublethal dose of deltamethrin was 25.5% (95% CI = 20.8–31.3%). Additionally, for both strains and all insecticides, statistical analyses revealed significant differences in blood-meal acceptance rates among mosquitoes exposed to non-insecticide-treated and insecticide-treated bottles (Fig. 3). Kisumu mosquitoes exposed to chlorfenapyr (*P* = 0.002, OR = 0.54, 95% CI = 0.39–0.75) and broflanilide (*P* < 0.001, OR = 0.34, 95% CI = 0.24–0.47) were approximately half as likely to feed as those in the control group. With deltamethrin, there was an 83% reduction in the likelihood of feeding (*P* < 0.001, OR = 0.17, 95% CI = 0.12–0.25). When mosquitoes from Tiassalé were exposed to chlorfenapyr (*P* < 0.001, OR = 0.45, 95% CI = 0.32–0.62) or broflanilide (*P* < 0.001, OR = 0.49, 95% CI = 0.36–0.68), the chance of feeding decreased by around 50%. The effect of deltamethrin, meanwhile, was even stronger in the Tiassalé strain (*P* < 0.001, OR = 0.20, 95% CI = 0.14–0.30) compared with broflanilide and chlorfenapyr.

### Effect on viable egg production and proportion of females with viable eggs

The number of viable eggs laid by Tiassalé strain exposed to all three insecticides varied significantly compared with control mosquitoes (Kruskal–Wallis H-test, *H* = 64.4, *df* = 4, *P* < 0.001). A more detailed comparison (proportion comparison test, with post hoc Holm adjustment) of the different treatments showed that Tiassalé strain exposed to a sublethal dose of chlorfenapyr or broflanilide showed a significant reduction in the average number of viable eggs per female, with respective averages of 13.67 (95% CI = 13.23–14.65, *P* < 0.0001) and 15.27 (95% CI = 10.5–20.04, *P* < 0.0001), compared with those exposed only to control bottles [acetone: 36.74 (95% CI = 31.04–42.43) egg/female and acetone + Mero: 35.6 (95% CI = 30.8–40.4) egg/female]. In contrast, again compared with the control groups, pre-exposure to deltamethrin LD_20_ showed no significant difference in the average number of viable eggs observed [mean = 26.03 (95% CI = 25.0–27.1) eggs/female, *P* = 0.09]. Similar results were observed for the Kisumu strain after exposure to the three insecticides. Furthermore, for the Tiassalé strain, exposure to broflanilide [13.79 (13.21–14.40) eggs/female, *P* < 0.0001] and chlorfenapyr (23.44 eggs/female, *P* = 0.0005) resulted in a statistically significant difference in the mean number of viable eggs compared with those exposed to deltamethrin (31.66 eggs/female) (Table 3).

**Table 3:** Effect of sublethal exposure of insecticides on the viable egg production of Kisumu and Tiassalé strains of *Anopheles gambiae* mosquitoes

Mosquito strain	Insecticide	Mosquito (*n*)	NVE	MNVE (95% CI)	*P*-value
Tiassalé	Broflanilide	116	1771	15.27 (10.5–20.04)	< 0.0001^*^
	Chlorfenapyr	118	1633	13.67 (13.23–14.65)	< 0.0001^*^
	Deltamethrin	91	2369	26.03 (25.05–27.11)	0.09
	Acetone	133	4887	36.74 (31.04–42.43)	-
	Acetone + Mero	135	4812	35.61 (30.31–40.91)	-
Kisumu	Broflanilide	134	1848	13.79 (13.21–14.40)	< 0.0001^*^
	Chlorfenapyr	135	3165	23.44 (22.62–24.30)	0.0006^*^
	Deltamethrin	79	2501	31.66 (30.38–32.86)	0.46
	Acetone	123	4799	39.01 (33.15–44.87)	-
	Acetone + Mero	120	4870	40.58 (34.72–46.43)	-

Fertility is the mean number of viable eggs per mosquito female. MNVE unit is eggs per female (eggs/female). *n* is the number of mosquitoes exposed. *NVE* number of viable eggs, *MNVE* mean number of viable eggs. *CI* confidence interval

^*^Statistically significant

Regarding viable egg production, results showed that broflanilide and chlorfenapyr at sublethal doses significantly reduced the rate of females with viable eggs in both strains, while deltamethrin had only a weak and insignificant effect (Table 4). In Tiassalé, the rate fell from 67.7 (59.0–75.5) and 63.7 (55.0–71.8) in control groups to 58.2% (95% CI = 47.3–68.4) for deltamethrin, and then to 41.5% (95% CI = 32.6–50.9) for chlorfenapyr and 27.6% (95% CI = 20.3–36.3) for Broflanilide. The difference of all these proportions was statistically significant (*χ*^2^ = 54.73, *df* = 4, *P* < 0.0001). In Kisumu, the values were also contrasting: 71.9% (95% CI = 63.1–79.3) for the control versus 64.6% (95% CI = 53.4–74.3) for deltamethrin, and 54.1% (95% CI = 45.4–62.5) and 41.0 (95% CI = 33.1–49.5) for chlorfenapyr and broflanilide. The Chi square test confirmed an overall difference (*χ*^2^ = 35,94, *df* = 4, *P* < 0.0001). For further analyses, pairwise comparisons revealed the following trends. In the Tiassalé strain, chlorfenapyr (Pearson *χ*^2^ = 17.66, *df* = 1, *P* < 0.0001) and broflanilide (Pearson *χ*^2^ = 31.24, *df* = 1, *P* < 0.0001) significantly reduced the rate of females with viable eggs compared with deltamethrin and also their control. Deltamethrin did not differ significantly from its control (Pearson *χ*^2^ = 3.18, *df* = 1, *P* = 0.07). In the Kisumu strain, only the comparison between chlorfenapyr (Pearson *χ*^2^ = 9.28, *df* = 1, *P* = 0.002) and broflanilide (Pearson *χ*^2^ = 19.06, *df* = 1, *P* < 0.0001) against their controls was significant (*P* < 0.05).

**Table 4:** Effect of sublethal exposure of insecticides on the: Proportion of females with viable eggs from Tiassalé and Kisumu strains of *Anopheles gambiae* mosquitoes

Mosquito strain	Insecticide	Mosquito (*n*)	FVE	Percentage (%) of FVE (95% CI)	*P*-value
Tiassalé	Broflanilide	116	32	27.6 (20.3–36.3)	< 0.0001^*^
	Chlorfenapyr	118	49	41.5 (33.0–50.5)	< 0.0001^*^
	Deltamethrin	91	53	58.2 (48.0–67.8)	0.07
	Acetone	133	90	67.7 (59.0–75.5)	-
	Acetone + Mero	135	86	63.7 (55.0–71.8)	-
Kisumu	Broflanilide	134	55	41.0 (33.1–49.5)	< 0.0001^*^
	Chlorfenapyr	135	73	54.1 (45.7–62.2)	0.002^*^
	Deltamethrin	79	51	64.6 (53.6–74.2)	0.31
	Acetone	123	90	73.2 (64.4–80.8)	-
	Acetone + Mero	120	83	69.2 (60.1–77.3)	-

*n* is the number of mosquitoes exposed. *FVE* number of mosquito females with viable eggs, *MNVE* mean number of viable eggs. *CI* confidence interval

^*^Statistically significant

## Discussion

This study assessed the sublethal effects of three insecticides – broflanilide (meta-diamide), chlorfenapyr (pyrrole) and deltamethrin (pyrethroid) – on the reproductive traits (longevity, blood-meal acceptance, viable egg production and proportion of females with viable eggs) of insecticide-susceptible Kisumu and insecticide-resistant Tiassalé populations of *An. gambiae* mosquitoes. Overall, we found a significant impact of sublethal doses (LD_20_) of all insecticides on all life history traits measured in both strains. However, broflanilide induced stronger and more sustained inhibitory effects on longevity, viable egg production and proportion of females with viable eggs compared with chlorfenapyr and deltamethrin, particularly in the pyrethroid-resistant Tiassalé strain. These findings suggest that sublethal insecticide exposure can substantially reduce mosquito reproductive potential and impair blood-feeding behaviour.

The LD_20_ values varied considerably across insecticides and mosquito strains examined in this study. For broflanilide and chlorfenapyr the LD_20_ values were notably low, indicating that these compounds exert biological effects at minimal doses, even in mosquito populations recognized to be resistant to multiple classes of insecticide. In contrast, the LD_20_ for deltamethrin was close to the diagnostic dose (12.6 µg/bottle) in the Tiassalé strain and much higher than that observed with the Kisumu strain, suggesting strong resistance to this insecticide in the Tiassalé population. Previous studies have similarly reported high levels of pyrethroid resistance, including deltamethrin, in *An. gambiae* Tiassalé populations, primarily mediated by *kdr* mutations and metabolic mechanisms [37, 43].

A significant reduction in longevity was observed with broflanilide in the Tiassalé population, whereas the effects of chlorfenapyr and deltamethrin were not statistically significant compared with the control group. Both broflanilide and chlorfenapyr caused a rapid decline in mosquito longevity during the first week post-exposure. The effect of broflanilide was more sustained, while for chlorfenapyr, daily mortality gradually stabilized and approached control levels after about 10 days. The marked effect of broflanilide on the longevity of the pyrethroid-resistant Tiassalé population likely reflects its mode of action. By targeting GABA-gated chloride channels, it disrupts key neurological functions and impairs muscle control, consequently reducing the mosquitoes’ ability to survive following exposure [50]. A study carried out in South Africa demonstrated that oxidative stress can significantly reduce the longevity of *An. gambiae* mosquitoes under laboratory conditions [51]. Field and laboratory studies in Burkina Faso [52], Benin [38] and Tanzania [18] have shown that broflanilide has long-lasting residual efficacy, with a mortality rate of ≥ 80% in wild populations of *An. gambiae* resistant to pyrethroids for up to 9–12 months after indoor spraying. This could disrupt the development of the *Plasmodium* parasite which takes 10–12 days in mosquitoes [53] and prevent malaria transmission. Chlorfenapyr, although a slow-acting metabolic insecticide, appears to have a partially attenuated effect on the longevity in the Tiassalé strain, as shown by the survival curves and Cox model. This pattern may reflect the intense agricultural activity and sustained insecticide pressure in Tiassalé, which could have driven specific genetic adaptations in local mosquito populations [3, 43, 54]. Indeed, chronic exposure of mosquitoes at the larval stage to agrochemical mixtures can induce increased tolerance to insecticides used in public health, promoting physiological adaptations such as cuticle thickening or gene expression modulation, which could limit the penetration or action of chlorfenapyr, particularly at sublethal doses [55, 56]. In the current study, deltamethrin exposure did not significantly affect the longevity of the pyrethroid-resistant Tiassalé strain but caused a significant reduction in the lifespan of the pyrethroid-resistant Kisumu. The absence of a significant sublethal effect of deltamethrin on the longevity of the Tiassalé strain likely reflects its high and multifaceted resistance to pyrethroids [37, 57, 58]. Deltamethrin LD_20_ might not induce sufficient physiological stress able to affect the lifespan of surviving mosquitoes, as reported in Tanzania were a surviving wild strain of *An. arabiensis* lived longer than the susceptible strain after both were exposed to deltamethrin and permethrin [59]. These results suggest that resistance status can substantially modulate the expression of sublethal effects on survival, particularly for insecticides with neurotoxic modes of action.

Reduced blood-feeding success represents an important sublethal outcome, as it directly affects mosquito fitness and vectorial capacity by limiting access to blood meals required for egg development and pathogen transmission. The current experiments show that deltamethrin had the largest impact on reducing blood meal acceptance in both Kisumu and Tiassalé strains, although the effects of chlorfenapyr and broflanilide were also statistically significant. Deltamethrin, a contact insecticide, leads to a knockdown effect in both strains, characterized by a rapid, temporary paralysis of exposed mosquitoes due to hyperexcitation of the neurons, which disrupts the lucidity of the mosquitoes. As a result, the mosquito’s ability to find and take a blood meal within 30 min after exposure to the sublethal dose could be significantly impaired. The knockdown effect of deltamethrin has been corroborated in several studies, which have attributed the knockdown resistance (*kdr*) mutation to resistance to deltamethrin. This mutation is characterized by an amino acid substitution in the voltage-gated sodium channel, the main target of deltamethrin [60–64]. The significant but less considerable effects of broflanilide and chlorfenapyr compared with deltamethrin show that, although they are slow-acting insecticides [17, 23], these insecticides begin to have an impact on mosquitoes from the very first moments of contact. However, it is necessary to extend the 30-min period after exposure to the sublethal dose for these three molecules, particularly for deltamethrin, to determine at what point the effects on blood-meal acceptance becomes insignificant.

Exposure to a sublethal dose of insecticides was associated with reductions in reproductive output, as reflected by lower proportions of females producing mature eggs and reduced numbers of viable eggs per female. For both Tiassalé and Kisumu strains, only broflanilide and chlorfenapyr significantly reduced viable egg production and proportion of females with viable eggs. This suggests a potential disruption of reproductive functions through alterations in neurohormonal regulation and oxidative phosphorylation, which are involved in oocyte maturation and embryonic development [65, 66]. Through its neuronal mode of action, antagonizing GABA receptors and inducing prolonged nerve inhibition, broflanilide may strongly disrupt the neural signalling pathways that regulate mosquito reproduction. This point is corroborated by studies conducted on *Spodoptera litura* and *Tuta absoluta*, which showed that sublethal exposures (LC_10_–LC_25_) to broflanilide significantly reduce fertility and fecundity in these insects [67, 68]. Additionally, chlorfenapyr, which inhibits ATP production in mitochondria [21], could have induced a significant energy deficit, affecting egg viability. These findings highlight the multifunctional potential of these insecticides beyond their immediate lethal effects. By also impairing key biological functions, they may contribute to a gradual decline in vector populations in areas with high insecticide resistance, ultimately influencing malaria transmission dynamics. However, the mechanisms underlying these effects remain unclear. Neurohormonal or metabolic disruptions induced by these insecticides may contribute to reduced viable egg production and a lower proportion of egg-laying females, both closely linked to fecundity and fertility, but this hypothesis requires confirmation through further investigation. It would also be of interest to explore whether the sublethal actions of these insecticides are not limited solely to the first post-exposure egg-laying period. The limited effect of deltamethrin on the viable eggs production and rate of females with viable eggs of susceptible Kisumu strain mosquitoes may be due to its relatively weak action in this context, especially since the mosquitoes were not exposed to lethal doses (near residual dose 0.022 µg/bottle). In the resistant Tiassalé strain, the reduced efficacy of deltamethrin is likely due to the presence of multiple resistance mechanisms [57, 69]. Although some studies suggest that pyrethroid resistance can be linked to decreased fecundity and/or fertility in *Ae. aegypti* and *Culex quinquefasciatus* [70–72], this effect does not appear to be consistently observed when directly assessing the impact of deltamethrin on these reproductive parameters.

The present study showed a sublethal exposure to insecticides inhibits key reproductive traits of surviving individuals of resistant *Anopheles* mosquitoes. Here, the sublethal exposure altered their longevity, blood-feeding behaviour, viable egg production and proportion of females with viable eggs in the insecticide-resistant Tiassalé strain. This underscores the importance of assessing the sublethal attribute of the active ingredients, in addition to the conventional indicators (i.e., mortality, blood-feeding and knock-down), when evaluating the efficacy of new vector control tools, included in ITNs or IRS. Several early experiments have demonstrated the sublethal effects of insecticides on the mosquito life-history traits and vectorial capacities for *Plasmodium* transmission [8, 73–77]. Sublethal effects of insecticides reported in the current study might pave the way for the strategic use of these compounds in areas of high resistance, for a truly comprehensive approach to combating malaria transmission. Broflanilide induced stronger sublethal effects on longevity, viable egg production and rate of females with viable eggs in both Kisumu and Tiassalé strains. Additionally, Broflanilide showed very low LD_20_ in the pyrethroid-resistant Tiassalé strain, therefore demonstrating high killing capacities against resistant vectors. Previous laboratory and field trials have shown the high efficacy and promising effect of Broflanilide against pyrethroid-resistant *Anopheles* mosquitoes in Benin, Burkina Faso, Tanzania and Ethiopia [15, 18, 38, 78–80]. Broflanilide may be recommended as an active ingredient for the development of new malaria vector control tools to more sustainable malaria prevention strategies.

This study has several limitations. Molecular species identification and contemporaneous resistance profiling were not conducted on the experimental cohorts. In addition, reproductive assays did not include virgin separation or egg hatch measurements. Consequently, the mechanisms underlying reduced reproductive output could not be directly assessed. Future studies incorporating controlled mating designs, egg viability assessments and species-specific analyses will be essential to more precisely characterize how sublethal insecticide exposure affects mosquito reproduction and population dynamics.

## Conclusions

This study highlights the significant effects of sublethal doses of broflanilide, chlorfenapyr and deltamethrin on key biological traits of both insecticide-susceptible and insecticide-resistant strains of *An. gambiae* mosquitoes. Results demonstrated that sublethal exposure (LD_20_) to the slow-acting insecticides broflanilide and chlorfenapyr gave a notable reduction in longevity and reproduction outcomes for the resistant Tiassalé strain, while deltamethrin had a more significant impact on blood meal acceptance. This suggests that sublethal doses, as well as being involved in the development of resistance, could also reduce mosquito vectorial capacity. Despite their limited lethal efficacy against resistant vectors, exposure to sublethal doses of broflanilide, chlorfenapyr and deltamethrin can have an impact, particularly on vector density and biting ability. The findings of the present study recommend including the effect of sublethal exposure on reproductive traits as indicators for evaluating the efficacy of vector control tools. Moreover, broflanilide is a promising active ingredient that could improve the effectiveness of malaria vector control tools.

## Data Availability

The datasets supporting the findings are either included within the manuscript.

## Abbreviations

CI: Confidence interval
GABA: Gamma-aminobutyric acid
GADM: Global Administrative Areas
GPIRM: Global Plan for Insecticide Resistance Management in Malaria Vectors
IRS: Indoor residual spraying
ITNs: Insecticide-treated nets
*kdr*: Knockdown resistance
LD_20_: Lethal dose 20%
QGIS: Quantum Geographic Information System
WHO: World Health Organization
